# Mini review: STING activation during non-alcoholic fatty liver disease

**DOI:** 10.3389/fnut.2023.1139339

**Published:** 2023-03-01

**Authors:** Honggui Li, Xinlei Guo, Eduardo Aquino, Chaodong Wu

**Affiliations:** Department of Nutrition, Texas A&M University, College Station, TX, United States

**Keywords:** non-alcoholic fatty liver disease, liver fibrosis, inflammation, stimulator of interferon genes, macrophage

## Abstract

Non-alcoholic fatty liver disease (NAFLD) is one of the most common chronic diseases serving as a major threat to human health. While the pathogenesis of NAFLD is multi-factorial, inflammation is considered a critical factor driving the development and progression of NAFLD phenotype, including liver fibrosis. As an essential mediator of innate immunity, stimulator of interferon genes (STING) functions to promote anti-viral immunity. Accumulating evidence also indicates that STING functions to promote the proinflammatory activation of several types of liver cells, especially macrophages/Kupffer cells, in a manner independent of interferon production. Over the past several years, a significant body of literature has validated a detrimental role for STING in regulating the pathogenesis of hepatic steatosis and inflammation. In particular, the STING in macrophages/Kupffer cells has attracted much attention due to its importance in not only enhancing macrophage proinflammatory activation, but also generating macrophage-derived mediators to increase hepatocyte fat deposition and proinflammatory responses, and to activate hepatic stellate cell fibrogenic activation. Both intracellular and extracellular signals are participating in STING activation in macrophages, thereby critically contributing to NAFLD phenotype. This mini review summarizes recent advances on how STING is activated in macrophages in the context of NAFLD pathophysiology.

## 1. Introduction

Non-alcoholic fatty liver disease (NAFLD), comprised of a spectrum of chronic liver diseases whose pathogenesis is not associated with alcohol consumption, is characterized by excessive fat deposition in hepatocytes (steatosis). Subjects with certain health conditions such as obesity, metabolic disease, and insulin resistance, are more prone to develop NAFLD. Indeed, epidemiological data indicate that the incidence of NAFLD in populations with obesity is increased by about sevenfold relative to that of general populations. While simple steatosis may be benign and have few or no symptoms, NAFLD progresses to non-alcoholic steatohepatitis (NASH), the advanced form of NAFLD, when the liver displays overt inflammatory damage. The diagnosis of NAFLD relies on medical history, a physical exam, and laboratory tests. However, liver histology is required to diagnose NASH. Healthy eating and lifestyle changes are recommended to manage NALFD, particularly hepatic steatosis. However, currently there is no effective treatment for NASH.

The outcomes of numerous basic research and translational studies have demonstrated that NASH is one of the most common causes of terminal liver diseases including hepatocellular carcinoma. Because of this, a significant number of investigations have focused on how inflammation arises and then triggers or exacerbates the development and progression of NAFLD under obese conditions. As a critical mediator in innate immunity, stimulator of interferon genes (STING) has been implicated to promote inflammation. This property enables STING to be involved in the pathogenesis of NAFLD. This mini review focuses on the recent advances in understanding STING activation in macrophages during the pathogenesis of NAFLD.

## 2. Role of STING in the pathogenesis of NAFLD

While studying DNA-binding agents for their anti-tumor properties, researchers synthesized and functionally characterized the effect of 5,6-dimethylxanthenone-4-acetic acid (DMXAA) in 1990 (1). Since then, the investigations concerning DMXAA actions have been extended to studying the regulation of macrophage activation as it related to anti-tumor immunology. In 1994, Perera et al. showed that DMXAA was able to induce a subset of lipopolysaccharide (LPS)-inducible genes within primary murine macrophages (2). Several years later, the lab where DMXAA was initially synthesized showed that DMXAA was capable of inducing interferon (IFN) expression (3), suggesting an effect of DMXAA on stimulating antiviral defensive responses in addition to its role in promoting the proinflammatory responses. Following these findings, numerous studies had focused on the mechanisms by which DMXAA induces IFN expression. In 2007, Roberts et al. demonstrated DMXAA as a novel and specific activator of the TANK-binding kinase 1 (TBK1)-IFN regulatory factor 3 (IRF-3) signaling pathway (4). A year later, the adaptor protein mediator of IRF3 activation (MITA), known as STING, was functionally validated to serve as a critical mediator of virus-induced IFN beta (IFNβ) expression (5). Since then, STING has been studied for its role in regulating innate immunity and the proinflammatory responses (6, 7).

The discovery of cyclic 2′,5′-GMP-AMP (cGAMP), generated by cGAMP synthase (cGAS) in response to cytosolic aberrant presence of double-stranded DNA (dsDNA), has drawn much attention to STING for its role in anti-viral immunity. While examining the effects of cGAMP, the studies by Guo et al. and Luo et al. showed interesting findings concerning macrophage activation. Specifically, treatment of bone marrow cell-derived macrophages (BMDMs) that were prepared from wild-type (WT) mice with exogenous cGAMP potentiated the effect of LPS on stimulating macrophage proinflammatory activation (8). However, in the absence of functional STING, treatment of mouse BMDMs with exogenous cGAMP significantly decreased the effect of LPS on stimulating macrophage proinflammatory activation (9). These findings led Luo et al. to postulate a role for STING in regulating inflammation as a key driving factor to promote the pathogenesis of NAFLD. Accordingly, Luo et al. performed the first study to examine STING expression in livers from patients with NAFLD and demonstrated a significant increase in STING expression in liver non-parenchymal cells (NPCs) relative to that in livers from subjects without NAFLD (9). Notably, using various mouse models involving adoptive transfer of STING-disrupted bone marrow cells to lethally irradiated WT mice or adoptive transfer of WT bone marrow cells to lethally irradiated STING-disrupted mice, the study by Luo et al. validated a deleterious role for STING-driven macrophage activation in promoting hepatic steatosis and inflammation, as well as liver fibrosis. The concept that STING-driven macrophage activation critically contributes to the development and progression of NAFLD or NASH with liver fibrosis is substantiated by the findings from the study by Wang et al. upon analyzing STING expression in liver monocyte-derived macrophages and Kupffer cells (10). Notably, using liver sections from human subjects with or without NAFLD, Wang et al. demonstrated that STING expression is significantly increased in liver sections from patients with NAFLD (10). More importantly, the intensities of STING expression in macrophages, particular monocyte-derived macrophages, were positively correlated with the severities of NASH and/or grades of liver fibrosis. To be noted, since the first study addressing STING promotion of NAFLD by Luo et al., a significant number of new studies have confirmed the importance of STING in NAFLD or NASH pathogenesis, as well as liver inflammation and related injuries (11–15), and provided novel insights into STING activation in macrophages, which are highlighted below.

## 3. Hepatocyte-driven STING activation

The earliest evidence concerning the role for STING in regulating liver inflammation and fibrosis may come from the results of the study by Petrasek et al. (16). Notably, global deficiency of IRF3, a transcription factor whose activation is induced in response to STING activation, protected mice from ethanol-induced liver inflammatory damage. Due to its role in activating IRF3 and related downstream events, STING was also studied for its role in ethanol-induced liver inflammation. Specifically, STING was detectable at high abundance in whole liver extracts and associated with phosphorylated IRF3. Moreover, STING disruption blunted the effect of thapsigargin, a non-competitive inhibitor of the sarcoendoplasmic reticulum Ca^2+^ ATPase, on activating (phosphorylating) IRF3 in mouse primary hepatocytes and alleviated the severity of ethanol-induced liver inflammation. Based on this, the authors speculated a role for STING in hepatocytes in promoting liver inflammation (16). A similar study by Iracheta-Vellve et al. also indicated a role for STING in hepatocytes in its association with IRF3 in the context of promoting carbon tetrachloride (CCl4)-induced hepatocyte apoptosis and liver fibrosis (17). Interestingly, in either of the two studies the researchers only provided the data concerning STING expression in whole liver extracts, but not hepatocytes, while a role for the STING in hepatocytes has been speculated. In contrast, increasing evidence from the studies involving both human subjects and rodent models indicates that STING expression in hepatocytes is likely at an undetectable level as this is supported by the data from immunoblots for STING expression in the isolated primary hepatocytes or liver immunohistochemistry staining for STING (9, 11, 14, 18). Because of this, the STING in macrophages, but not hepatocytes, appears to be primarily responsible for the regulation of liver inflammation. In addition, macrophage STING activation is attributable to, in large part, hepatocyte-derived mediators. This is particularly true under conditions with hepatic steatosis; given that hepatocyte fat deposition is an event that commonly precedes inflammation.

In terms of how hepatocyte fat deposition causes or exacerbates liver inflammation, multiple studies have demonstrated the importance of palmitate as a key component of fats in triggering or enhancing hepatocyte proinflammatory responses. For instance, the study by Nakamura et al. showed that palmitate induced hepatocyte JNK activation, at least through promoting the production of mitochondrial dysfunction-associated reactive oxygen species (ROS) (19). Since palmitate-treated hepatocytes revealed increased production of proinflammatory mediators that are capable of enhancing macrophage proinflammatory activation (20, 21), it is conceivable that excessive fat deposition-associated hepatocyte mediators function to stimulate macrophage proinflammatory activation and this process involves STING. This, indeed, was validated by the findings from the study by Yu et al. Initially, Yu et al. verified that STING was highly expressed in liver Kupffer cells and was nearly undetectable in hepatocytes of WT mice while showing that global STING disruption protected mice from either high-fat die (HFD)-induced NAFLD or methionine- and choline-deficient diet (MCD)-induced NASH. This led the researchers to validate hepatocyte-derived mitochondrial DNA (mtDNA) as a key mediator that functions to stimulate the STING in Kupffer cells, as a result of cGAS activation and cGAMP production, and increase the expression of proinflammatory cytokines in Kupffer cells. This in turn accounts for liver inflammation. In addition, STING promotion of the expression of proinflammatory cytokines in Kupffer cells was largely independent of IRF3. However, it remains an important question whether hepatocyte-derived mtDNA is sufficient to trigger or exacerbate NAFLD or NASH phenotype while stimulating STING in liver Kupffer cells and promoting proinflammatory cytokine expression.

While increased mtDNA production is a hallmark of hepatocyte mitochondrial dysfunction/stress, excessive fat deposition is considered to trigger or exacerbate hepatocyte mitochondrial dysfunction. Indeed, the study by Yu et al. hinted at a link between excessive fat deposition and hepatocyte mitochondrial dysfunction as this was evidenced by the finding that hepatocyte mtDNA production is increased in mice fed an HFD. However, palmitate, proposed as a primary fatty acid that functions to induce hepatocyte mitochondrial dysfunction, is a hydrolytic product of VLDL, whose secretion by hepatocytes is not necessarily elevated during hepatic steatosis. Considering this, aberrant subcellular deposition of fatty acids must exist in hepatocytes and account for, to a certain extent, hepatocyte mitochondrial dysfunction and mtDNA production during hepatic steatosis. This notion is supported by the findings of a recent study investigating the role for hepatocyte adenosine kinase (ADK) in regulating fat deposition and liver inflammation. In that study, Li et al. showed that hepatocyte-specific ADK overexpression caused excessive fat deposition in hepatocytes, which was characterized by increased mitochondrial membrane accumulation of multiple bis-monoacylglycerol phosphate and lysophosphatidylcholine species and significantly decreased membrane levels of tetralinoleoyl cardiolipin (18:2-18:2-18:2-18:2), indicating increased oxidative stress and mitochondrial dysfunction (22). The latter was accompanied with increased hepatic mtDNA production and increased STING activation in liver macrophages (22). As such, intracellular accumulation of fats in excessive amount is one of the primary causes of increased production of hepatocyte mtDNA, which in turn functions to stimulate macrophage cGAS-cGAMP signaling and STING activation.

During liver injury in the absence of excessive fat deposition, hepatocyte production/release of mtDNA is increased and causes STING activation, in response to increased cGAS-cGAMP signaling, in liver macrophages/Kupffer cells (13). Specifically, the study by Chen et al. showed that hepatocyte-specific deletion of Sam50, a key component of the sorting and assembly machinery (SAM) necessary for the assembly of β-barrel proteins in the mitochondrial outer membrane, induced cardiolipin-dependent mitochondrial membrane remodeling to trigger mtDNA release. The latter was accompanied by increased STING amount and signaling in liver cells including macrophages, which in turn accounted for increased liver inflammatory injury. Similarly, the study by Liu et al. showed that the X-box binding protein 1 (XBP1) in hepatocytes was linked to macrophage STING activation through hepatocyte-derived mtDNA (23).

## 4. Extrahepatic mediator-stimulated STING activation

While the interplays among liver cells are key to the pathogenesis of NAFLD, extra-hepatic tissues have been shown to also regulate the development and progression of hepatic steatosis and inflammation as this is highlighted by the multiple-hit theory for NAFLD. For instance, under obese conditions, various mediators associated with adipose tissue dysfunction participate in the initiation and progression of NAFLD phenotype. These mediators include fatty acids, adipokines, and cytokines (24), many of which have been implicated to trigger or enhance liver inflammation through their actions on both hepatocytes and liver NPCs (24). It is worth noting that intestinal inflammation is increased not only under obese conditions, but also under conditions of lipoatrophy or absence of abdominal fat as this is supported by outcomes from mice fed an obesogenic/pro-NAFLD HFD or MCD diet (25–28). In particular, MCD-fed mice displayed increased intestinal inflammation that was correlated with corresponding degrees of liver inflammation while showing almost no abdominal fat. This suggests that intestinal inflammation could be more important than white adipose tissue inflammation in terms of triggering or exacerbating liver inflammation under certain conditions, e.g., lipoatrophy.

As it is extensively reviewed elsewhere, gut dysbiosis is a key factor contributing to NAFLD pathogenesis (29–32). Of note, “leaky gut” is shown to increase the delivery of nutrients and proinflammatory mediators to the liver to promote NAFLD phenotype. This role of gut-derived mediator(s) is well exemplified by the effect of bacterial DNA on promoting liver inflammation in the context of STING involvement in the development of liver inflammation (12). Initially, Luo et al. validated that gut microbial DNA-containing extracellular vesicles (mEVs) were translocated in the circulation and metabolic tissues including the liver in both HFD-fed WT mice and obese humans relative to their respective control. This type of mEVs is shown to enhance liver inflammation in mice primarily through stimulating STING activation in liver cells including hepatocytes. The same outcome could be expected for liver macrophages, whose response to mEVs in terms of STING activation, however, was not examined. It is worth noting that liver CRIg+ macrophages were shown to exert a unique function of clearing intestinal mEVs from circulation, thereby protecting against liver inflammation. As substantial evidence, treatment of chow diet-fed CRIg-deficient mice with obese mEVs significantly increased the abundance of liver cytokines including interleukin 1 beta and tumor necrosis factor alpha relative to control-treated CRIg-deficient mice. This effect of mEVs was significantly lighter in WT mice, and was due to, in large part, the less activation of STING pathway in the liver. Based on these findings, it is conceivable that microbial DNA, when translocated to the liver, is capable of stimulating liver inflammation in a manner involving STING. While it remains debatable if normal hepatocytes express STING, it is very likely that the STING in liver macrophages can be activated by microbial DNA and account for liver inflammation.

## 5. Conclusion

**FIGURE 1:**
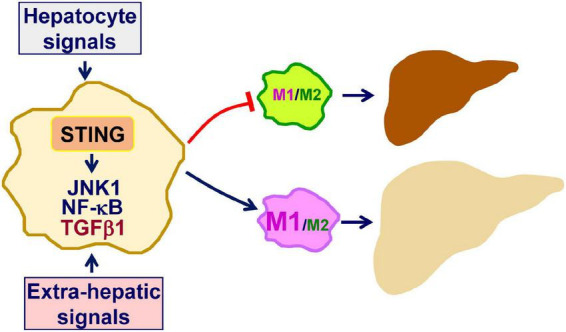
Macrophage STING promotes the pathogenesis of NAFLD. As a mediator of innate immunity, stimulator of interferon genes (STING) also exerts a crucial role in regulating inflammation. Within macrophages, double-stranded DNA (dsDNA) generated in response to mitochondrial dysfunction or endoplasmic reticulum stress activates cyclic GMP-AMP (cGAMP) synthase (cGAS) to increase cGAMP production. The latter, in turn, activates STING to promote macrophage proinflammatory activation. Inside the liver, hepatocytes are a key source that releases dsDNA including mitochondrial DNA and fragment DNA when under stress conditions. This, in turn, acts through cGAS-cGAMP signaling to activate the STING in macrophages. In addition, extra-hepatic mediators, i.e., gut-derived microbial DNA, exert a similar function in terms of activating macrophage STING in the liver. When activated, STING-stimulated macrophage activation promotes the development and progression of hepatic steatosis and inflammation. See text for details.

Inflammation not only underlies the pathogenesis of NAFLD, but also drives the progression of simple hepatic steatosis to NASH (9, 10, 21, 33–36). In terms of regulating inflammation, STING, an essential mediator of innate immunity, has also been implicated to exert a crucial role in promoting macrophage proinflammatory activation in a manner independent of IFNβ production (11, 17). Within the liver, macrophage STING can be activated by increased cGAS-cGAMP signaling in response to intracellular dsDNA due to mitochondrial stress or endoplasmic reticulum (ER) stress (14, 23). Moreover, under stress conditions, e.g., excessive fat deposition in hepatocytes, macrophage STING can be activated by increased cGAS-cGAMP signaling in response to exogeneous dsDNA, e.g., mtDNA, derived from hepatocytes (11, 16, 17, 22). This paracrine effect of hepatocyte-driven mediators on macrophage STING activation exemplifies dysregulation of crosstalk between macrophages and hepatocytes in the context of promoting the pathogenesis of NAFLD/NASH. In addition, during obesity, gut-derived microbial DNA can also be translocated to the liver and is expected to activate macrophage STING through cGAS-cGAMP signaling (12). The combined effects of hepatic and extrahepatic mediators on activating STING lead to enhanced macrophage proinflammatory activation, which in turn promotes liver inflammation and causes dysregulation of hepatocyte fat metabolism to trigger or exacerbate hepatic steatosis. The latter is attributable to, in large part, the paracrine effects of macrophage-driven mediators including proinflammatory cytokines on hepatocytes as reviewed elsewhere. As summarized in Figure 1, the STING in macrophages plays a central role in the pathogenesis of NAFLD. Accordingly, inhibiting STING in liver macrophages/Kupffer cells may be a viable approach for the management of NAFLD/NASH.

## Author contributions

HL, XG, and EA wrote in part of the manuscript. CW came up the concept and finalized the manuscript. All authors contributed to the article and approved the submitted version.
